# Echocardiographic windows in emergency departaments

**DOI:** 10.1186/2036-7902-6-S1-A10

**Published:** 2014-01-31

**Authors:** A Oviedo-García, M Algaba-Montes, J Lopez-Libano, JM Alvarez-Franco, N Diaz-Rodriguez, A Rodriguez-Lorenzo, A Segura Grau

**Affiliations:** 1 Emergency Department, Valme Hospital, Seville, Members of the Working Group of Ultrasound SEMES_Andalucía and SEMERGEN, Spain; 2 Critical Care Department, Miramar Hospital, Mallorca, Member of the Working Group of Ultrasound SEMERGEN, Spain; 3 Emergency Department, IB-Salut, Ibiza, Member of the Working Group of Ultrasound SEMERGEN, Spain; 4 Primary Care, Barbadás Primary Care Center, Ourense. Member of the Working Group of Ultrasound SEMERGEN, Spain; 5 Radiology Department, Perpetuo Socorro Hospital, Vigo, Member of the Working Group of Ultrasound SEMERGEN, Spain; 6 MFYC, Médico ecografista en Centro Diagnostico Ecográfico y en Unidad de ecografía general del Hospital San Francisco de Asis, Madrid, Spain

## Background

in Spain the skills of professionals of Emergency departament (ED) in the process of echocardiography have been discussed for decades. The current scientific evidence supports the use of echocardiography by the Emergency physicians (EP) in a resounding manner, due to its speed, agility and safety for the patient, by providing an early diagnosis of serious or potentially serious diseases. On the other hand, the use of ultrasound extends to almost all specialities (cardiologists, urologists, gynecologists, gastroenterologists…) in such a way that it already speaks of "Ultrasound as the stethoscope for the future”. ED doctors cannot be left behind.

## Objective

Training forwards the handling and diagnosis of the echocardiographic techniques among professionals in the ED services, and promoting their use on the basis of the advantages that this presents, due to its characteristics of safety, efficiency and safety for the patient.

## Patients and methods

The use of echocardiography, together with its different Windows and projections in critical care and ED services. We used a Sonosite M-Turbo ultrasound, equipped with P21 between 1 and 5 MHz probe.

## Results

Echocardiography is a technique of non-invasive diagnosis that uses ultrasonic waves to create images of the heart. As we know, the ultrasonic waves do not go through the hard tissues (bones), nor air (lungs), so in order to access the heart, they must penetrate through the between, on, and below (Windows). From these windows, the heart can be studied in three orthogonal cuts: longitudinal, transversal and axial. The view that we will consider are: Parasternal long-axis, short-axis, Apical four and five chambers and Subcostal four chambers.

## Conclusion

to **i**ncorporate echocardiography in ED decreases overall care times, therefore ED doctors are more effective, efficient and dynamic in the management of "time-dependent" emergencies, providing greater clinical safety.
